# MicroRNA-146b protects kidney injury during urinary tract infections by modulating macrophage polarization

**DOI:** 10.1128/mbio.02094-23

**Published:** 2023-11-01

**Authors:** Changying Wang, Hongyan Cheng, Fenglian Yan, Hui Zhang, Junfeng Zhang, Chunxia Li, Mingsheng Zhao, Dongmei Shi, Huabao Xiong

**Affiliations:** 1 Institute of Immunology and Molecular Medicine, Jining Medical University, Jining, China; 2 Jining Key Laboratory of Immunology, Jining Medical University, Jining, China; 3 Cheeloo College of Medicine, Shandong University, Jinan, China; 4 Department of Dermatology and Laboratory of Medical Mycology, Jining No. 1 People’s Hospital, Jining, Shandong, China; Columbia University, New York, New York, USA; Baylor College of Medicine, Houston, Texas, USA

**Keywords:** acute kidney injury, macrophage differentiation, urinary tract infections, uropathogenic *Escherichia coli*

## Abstract

Urinary tract infections (UTIs) caused by uropathogenic *Escherichia coli* (UPEC) can lead to severe kidney injury. However, the molecular mechanisms underlying the pathological process of kidney injury are still incompletely understood. In the present study, we demonstrate that microRNA-146b (miR-146b) deficiency aggravates kidney injury during UTIs caused by UPEC. In a mouse kidney infection model utilizing urosepsis isolate CFT073, we found that miR-146b expression significantly increased in the early stages of UPEC infection. Also, miR-146b-deficient mice displayed exacerbated inflammation in the kidney injury with severe M1 macrophage infiltration. Additionally, the results showed that miR-146b targeted interferon regulatory factor 5-regulated M1 macrophage polarization during UTIs. The results suggested that miR-146b contributed significantly to the control of kidney damage during UTIs, highlighting that miR-146b might be used as a novel therapeutic target for treating kidney injury during UTIs.

**IMPORTANCE:**

Kidney injury during acute urinary tract infections (UTIs) caused by uropathogenic *Escherichia coli* (UPEC) is an important public health problem. However, how kidney injury develops during UPEC infection is still unclear. Although antibiotic therapy is currently an effective treatment for UTI, it cannot avoid kidney injury. MicroRNAs have gained extensive attention as essential molecules capable of regulating the autoimmune response. Among these, microRNA-146b (miR-146b) is involved in regulating inflammatory responses. In the present study, we demonstrated that miR-146b played an essential role in the development of kidney injury during UTIs caused by UPEC. The results showed that miR-146b may suppress M1 macrophage polarization and alleviate acute kidney injury. Furthermore, the miR-146b activator, agomir, in order to upregulate miR-146b, was effective in treating kidney damage by inhibiting the activation of M1 macrophages. In conclusion, our findings elucidated the mechanisms by which miR-146b alleviated kidney injury induced by UTIs, shed new light on the relationship between microRNA and bacterial infection, and provided a novel therapeutic target for treating this common bacterial infection.

## INTRODUCTION

Urinary tract infections (UTIs) are commonly caused by uropathogenic *Escherichia coli* (UPEC). UTIs are categorized into lower urinary tract (e.g., cystitis) and upper urinary tract (e.g., pyelonephritis) infections based on the urinary tract structures. Once UTIs become severe, UPEC enters the bloodstream and causes sepsis (1, 2). Many patients experience recurrent or persistent UTIs within 12 months of the primary infection. Recurrent or persistent infections often develop into chronic pyelonephritis, causing long-term distress in patients (3). Pyelonephritis is classified as an upper UTI and can be divided into acute and chronic pyelonephritis, which can permanently damage the structures and functions of the kidneys (2, 3). Pyelonephritis or even symptomatic bacteriuria may cause severe complications in pregnant women, including low birth weight and preterm delivery (4).

Currently, antibiotic therapy is the most effective approach for treating UTI (3). UPEC can invade bladder epithelial cells and form biofilm-like intracellular bacterial communities or quiescent intracellular reservoirs to evade immune cell recognition and antibiotics, often leading to the reoccurrence of UTIs (3). However, the pathogenesis of bacterial colonization in infected kidneys is less known. Currently, it is known that multiple virulence factors of UPEC, such as type 1 pili, P pili, flagella, α-hemolysin, and cytotoxic necrotizing factor 1, are associated with adherence to kidney epithelium in some hosts (3, 5). Furthermore, antibiotics are proving ineffective in reducing the damage caused by UTIs due to the emergence of drug-resistant strains and intestinal disorders in patients, indicating an urgent need for developing new therapeutic approaches for UTIs (3, 6).

When UTIs occur, UPEC activates host immune responses, with many types of immune cells infiltrating into inflamed tissues. Immune cells, such as macrophages, neutrophils, natural killer (NK) cells, dendritic cells (DCs), and T cells, often function differently in UTIs. Innate immune responses play a major role in acute UTIs. Innate immune cells, including macrophages and neutrophils, are responsible for clearing bacteria (7–9). Neutrophils migrate into the uroepithelium during acute UTIs and are directed by macrophages, which are the main antigen-presenting cells in the mouse bladder during UTIs (10, 11). Macrophages can be of two types in inflamed tissues: resident and recruited macrophages. Inflammatory monocytes are recruited to infected tissues, and the cells differentiate into M1-type macrophages during pathogen invasion (11). Generally, macrophages are of two types: M1- and M2-type macrophages. M1 macrophages promote inflammatory responses, and M2 macrophages play a major role in pro-tumor functions, proliferation, and tissue repair (11–14). Previous studies showed that kidney damage was due to M1 macrophages (15). Thus, understanding the mechanisms of M1 macrophage differentiation may provide a theoretical basis for treating kidney injury.

MicroRNAs (miRNAs) are endogenous RNAs that regulate gene expression by targeting messenger RNA (mRNA) and post-transcriptional modifications. The nucleotide sequences of miRNAs comprise 20‒25 nucleotides, and miRNAs have high tissue specificity. miRNAs can bind to the 3′ end of mRNA to inhibit mRNA translation and regulate gene expression. In the human genome, approximately one-third of the human genes are regulated by miRNAs. miRNAs regulate developmental and physiological processes due to their unique features, including energy and lipid metabolism, fat formation, and body weight regulation (16, 17).

When human monocytes were stimulated with bacterial lipopolysaccharide (LPS), three miRNAs (miR-146a/b, miR-132, and miR-155) were significantly upregulated among 200 miRNAs (17). Furthermore, miR-146 includes miR-146a and miR-146b, which are coded by *MIR146A* and *MIR146B*, respectively. Although the two miRNAs are encoded by different genes, their mature sequences differ by only two nucleotides. Hence, miR-146a and miR-146b may have similar biological functions (17, 18). MiR-146a is mainly involved in the nuclear factor-κappa B signaling pathway (19). miR-146b expression is considerably altered after stimulation of human monocytes with bacterial LPS. Interleukin (IL)-10 can enhance miR-146b expression in human monocytes, thereby suppressing the activation of the Toll-like receptor 4 signaling pathway. MiR-146b can also target interferon regulatory factor 5 (IRF5) to inhibit the activation of M1 macrophages and improve colitis *in vivo* (18, 20). In a model of schistosomiasis infection, liver damage is related to the activation of M1 macrophages targeted by miR-146b (21, 22). In fever with thrombocytopenia syndrome, miR-146b can promote the differentiation of M2 macrophages, inhibiting the adaptive immune response (23). However, whether miR-146b is involved in UTIs caused by UPEC is still unclear. Thus, the present study aimed to explore how miR-146b affected M1/M2 differentiation in acute UTIs.

## RESULTS

### MiR-146b is highly expressed in the kidneys during UTIs

**FIG 1:**
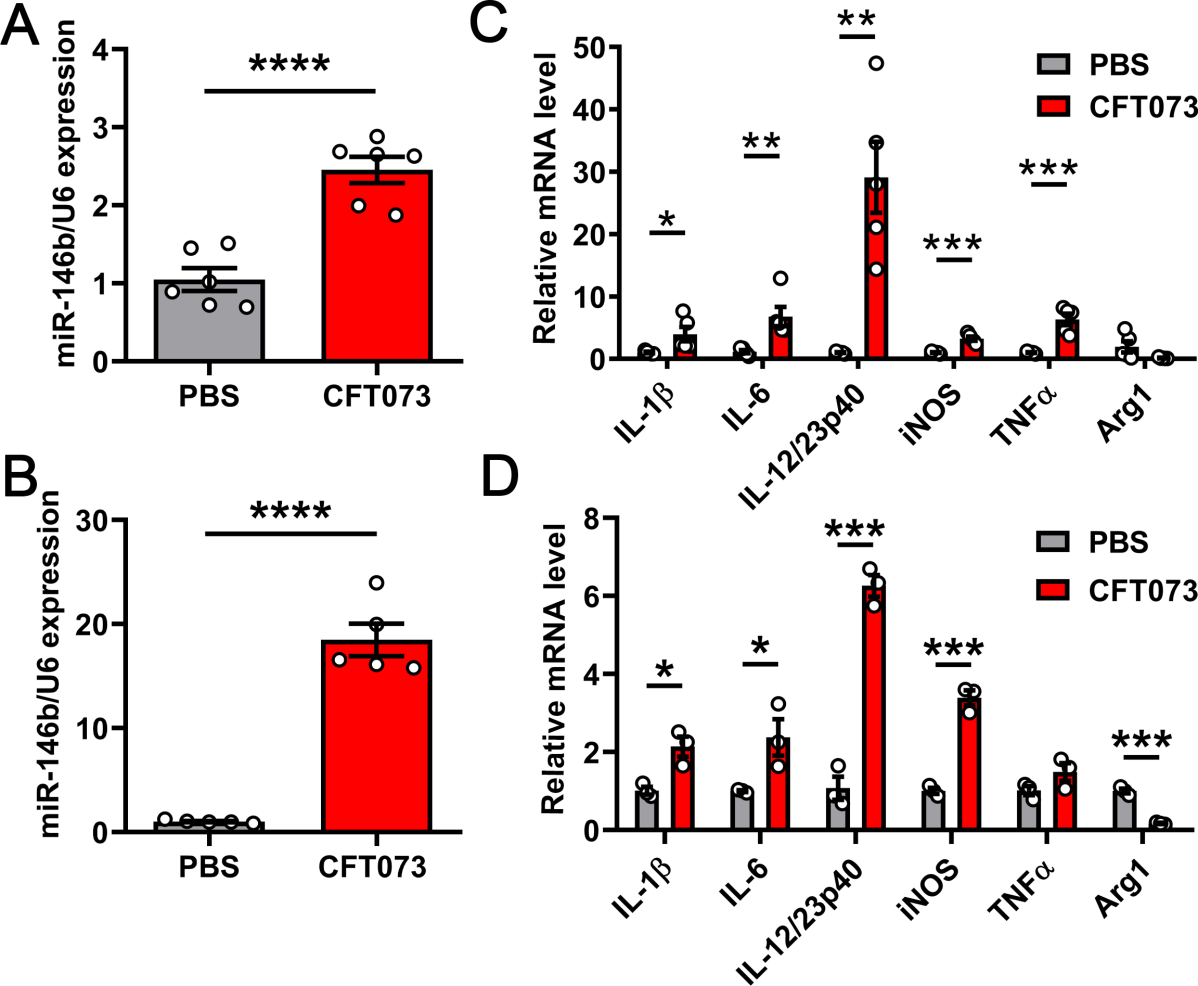
UPEC induced miR-146b expression in an acute UTI mouse model. (**A and C**) Female WT mice were inoculated intraurethrally using 1 × 10^9^ CFU of CFT073 twice at 3-h intervals. The mice were injected with an equal volume of phosphate-buffered saline (PBS) as a control. Quantitative polymerase chain reaction (qPCR) analysis of mRNA levels for miR-146b and inflammatory cytokines in infected kidneys after 24 h. (**B and D**) BMDMs from C57BL/6 mice were stimulated with CFT073 (multiplicity of infection = 0.01) for 6 h. qPCR analysis of mRNA levels for miR-146b and inflammatory cytokines in infected BMDMs. Data are expressed as mean ± SEM. Data were compared using the *t*-test. ^*^P < 0.05; ^**^P < 0.01; ^***^P < 0.001; ^****^P < 0.0001. All experimental data were collected in at least three independent experiments.

A previous study demonstrated that the level of miR-146b expression in the liver significantly increased and that miR-146a/b could protect the liver from damage when schistosomiasis infection occurred (21). Furthermore, miR-146b modulated the differentiation of M1 macrophages in colitis (20). We extended this study to the mouse model of UTIs, infected wild-type (WT) mice, and mouse bone marrow-derived macrophages (BMDMs) with CFT073 to further investigate the functions of miR-146b in other tissues. Quantitative polymerase chain reaction (qPCR) analysis showed that miR-146b-5p expression in the kidney and BMDMs significantly increased after infection with CFT073 (Fig. 1A and B). We also detected the levels of miR-146b-3p, miR-146a-5p, and miR-146a-3p using qPCR and found that their levels in the kidney slightly changed (Fig. S1A through C). A previous study reported that mature miR-146b-5p acted as a guide strand and mature miR-146b-3p functions acted as a passenger strand (18). Thus, we used miR-146b to represent miR-146b-5p throughout the study. We examined the expression of some representative inflammatory cytokines [IL-1β, IL-6, IL-12, tumor necrosis factor α (TNFα), inducible nitric oxide synthase (iNOS), and arginase 1 (Arg1)] to further investigate the role of miR-146b in the inflammation of kidneys and BMDMs infected with CFT073. The mRNA expression levels of *IL-1β*, *IL-6*, *IL-12*, *TNFα*, and *iNOS* increased. However, *Arg1* expression levels decreased (Fig. 1C and D). Hence, the results suggested that miR-146b was strongly associated with the inflammation caused by CFT073.

### Lack of miR-146b aggravates kidney damage

**FIG 2:**
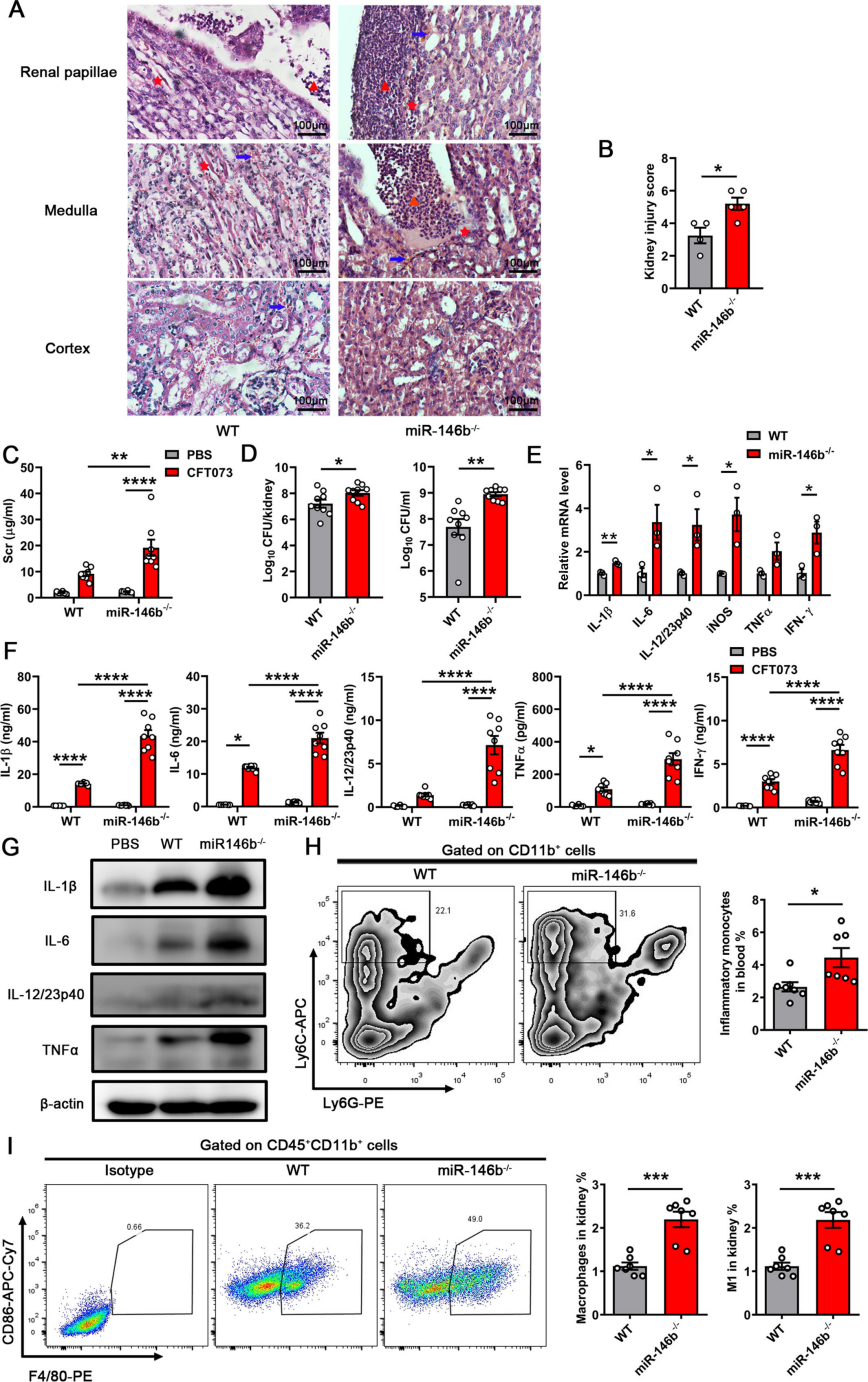
MiR-146b deficiency enhanced kidney injury caused by CFT073. Female WT and miR-146b^−/−^ mice were inoculated intraurethrally using 1 × 10^9^ CFU of CFT073 twice at 3-h intervals. The control for CFT073-infected mice was injected with an equal volume of phosphate-buffered saline (PBS) into WT mice. (**A**) Representative images of H&E staining of kidney tissues after 24 h. Arrows indicate serious hemorrhage, triangular symbols indicate inflammatory cell infiltration, and asterisks indicate renal papillae injury and protein casts. Scale bar, 100 µm. (**B**) Histological scores are shown in the right panel. (**C**) Serum creatinine (Scr) concentrations upon sacrifice 24 h after infection. (**D**) Tissue colony counts for kidney tissues (left) and urine (right) displayed as CFU. (**E-G**) qPCR analysis of mRNA levels, ELISA, and WB tests of inflammatory cytokines in infected kidneys after 24 h. (**H and I**) Representative flow cytometry plots and quantification of inflammatory monocytes in blood and macrophages in kidneys after 24 h. The data were expressed as the mean ± SEM. The *t*-test was used to compare between the groups (**B, D, E, G and I**). A one-way analysis of variance was used to compare the groups (**C and F**). ^*^P < 0.05; ^**^P < 0.01; ^***^P < 0.001; ^****^P < 0.0001. All experimental data were performed in triplicate.

We infected WT and miR-146b-deficient (miR-146b^−/−^) mice with CFT073 to further investigate the functions of miR-146b in kidney injury. Hematoxylin and eosin (H&E) staining showed that kidney damage was aggravated in the absence of miR-146b. Aggravated tissue hemorrhage and increased inflammatory cell infiltration are depicted by the identifiers in Fig. 2A; Fig. S2A. Furthermore, miR-146b deficiency increased the pathology scores for kidneys after being infected by CFT073 (Fig. 2B). Kidney function was impaired, as evidenced by increased levels of serum creatinine in miR-146b^−/−^ mice infected by CFT073 (Fig. 2C). Notably, the immunohistochemical analysis of the kidneys of mice infected with CFT073 showed that neutrophil gelatinase-associated lipocalin (NAGL) expression was higher in miR-146b^−/−^ mice (Fig. S2B). In miR-146b deficiency, the bacterial colonization in the kidney and urine was elevated (Fig. 2D). Consistent with the results, the mRNA expression levels of related inflammatory genes, including *IL-1β*, *IL-6*, *IL-12*, *iNOS*, *TNFα*, and *IFN-γ*, were found to be higher in miR-146b^−/−^ mice than in WT mice (Fig. 2E). The mRNA expression levels were also correlated with the increased protein levels of IL-1β, IL-6, IL-12, TNFα, and IFN-γ in the kidneys, as determined using the enzyme-linked immunosorbent assay (ELISA) (Fig. 2F). The results were also confirmed by western blot (WB) (Fig. 2G). We detected M1 macrophages in the kidney and inflammatory monocytes in blood infected with CFT073 using flow cytometry because miR-146b regulated the differentiation of M1 macrophages (20, 21). The results showed that the proportions of M1 macrophages and inflammatory monocytes were significantly higher in miR-146b^−/−^ mice than in WT mice (Fig. 2H and I). Meanwhile, other immune cells, including neutrophils in the blood, neutrophils, NK cells, DCs, B cells, and T cells in the kidney, showed little change during this process (Fig. S2C through J). These results indicated that miR-146b deficiency aggravated kidney inflammatory damage, which might be related to M1 macrophage infiltration modulated by miR-146b.

### MiR-146b-regulated macrophages are involved in kidney injury

**FIG 3:**
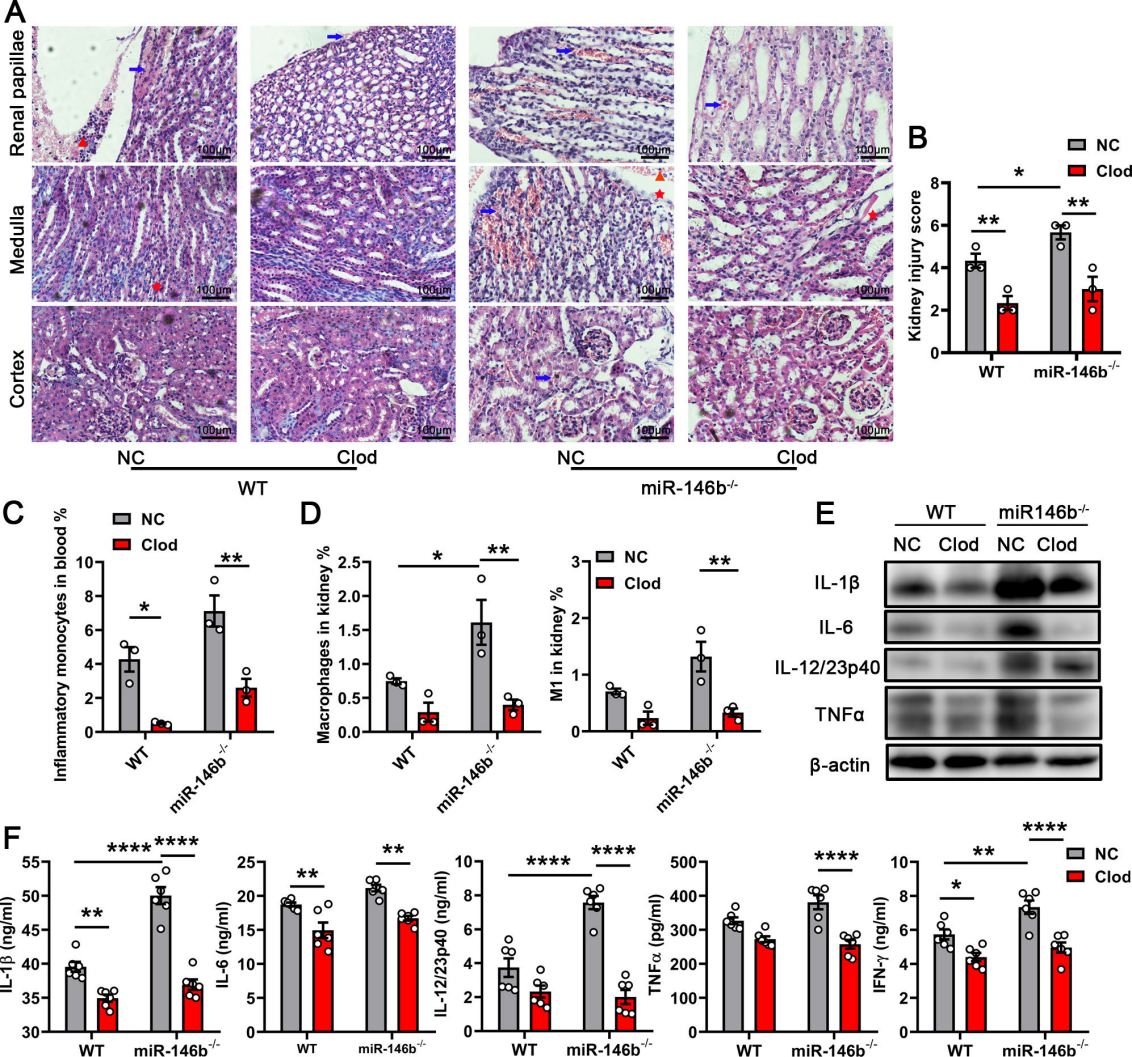
Macrophage elimination attenuated kidney injury caused by miR-146b deficiency. The mice were treated intravenously with Clod liposomes or NC liposomes for 24 h and subsequently infected with CFT073 for 24 h. (**A**) Representative images of H&E staining of kidney tissues. Arrows indicate serious hemorrhage, triangular symbols indicate inflammatory cell infiltration, and asterisks indicate renal papillae injury and protein casts. Scale bar, 100 µm. (**B**) Histological scores are shown in the right panel. (**C and D**) Quantification of inflammatory monocytes in blood and macrophages in kidneys after 24 h. (**E and F**) WB and ELISA results of inflammatory cytokines in the infected kidneys after 24 h. The data were expressed as the mean ± SEM. A one-way analysis of variance was used to compare the groups. ^*^P < 0.05; ^**^P < 0.01, ^****^P < 0.0001. All experimental data were collected in at least three independent experiments.

Our previous study showed that the damage caused by CFT073 was related to the addition of M1 macrophages (15). We used clodronate (Clod) liposomes to eliminate macrophages and control (NC) liposomes as a control to investigate if miR-146b also regulated macrophages to trigger kidney damage. Mice were infected with CFT073 for 24 h. We found that the depletion of macrophages in miR-146b-deficient mice reduced kidney damage caused by CFT073. As shown in Fig. 3A; Fig. S3A, the hemorrhage and inflammatory infiltration in mice were alleviated after treatment with Clod liposomes. Some inflammatory cells were observed in the kidneys infected with CFT073 even after treatment with Clod liposomes, likely due to the gradual removal of inflammatory cells (Fig. 3A; Fig. S3A). Pathology scores were taken and found to be reduced after eliminating macrophages with Clod liposomes in CFT073-infected kidneys (Fig. 3B). Expression of NAGL was also reduced in mice treated with Clod liposomes, which indicated that the macrophages were related to kidney injury during UTIs (Fig. S3B). Meanwhile, the percentages of inflammatory monocytes in the blood and macrophages, especially M1 macrophages, in the infected kidneys decreased after treatment with Clod liposomes in both WT and miR-146b^−/−^ mice (Fig. 3C and D). The levels of IL-1β, IL-6, and TNFα were also decreased in the infected kidneys of WT and miR-146b^−/−^ mice treated with Clod liposomes. However, the level of IL-12 showed little change in the WT group (Fig. 3E and F). The aforementioned results indicated that miR-146b deficiency promoted stronger kidney injury, suggesting that miR-146b modulated macrophages, resulting in kidney damage.

### MiR-146b targets IRF5 in regulating M1 macrophage polarization

**FIG 4:**
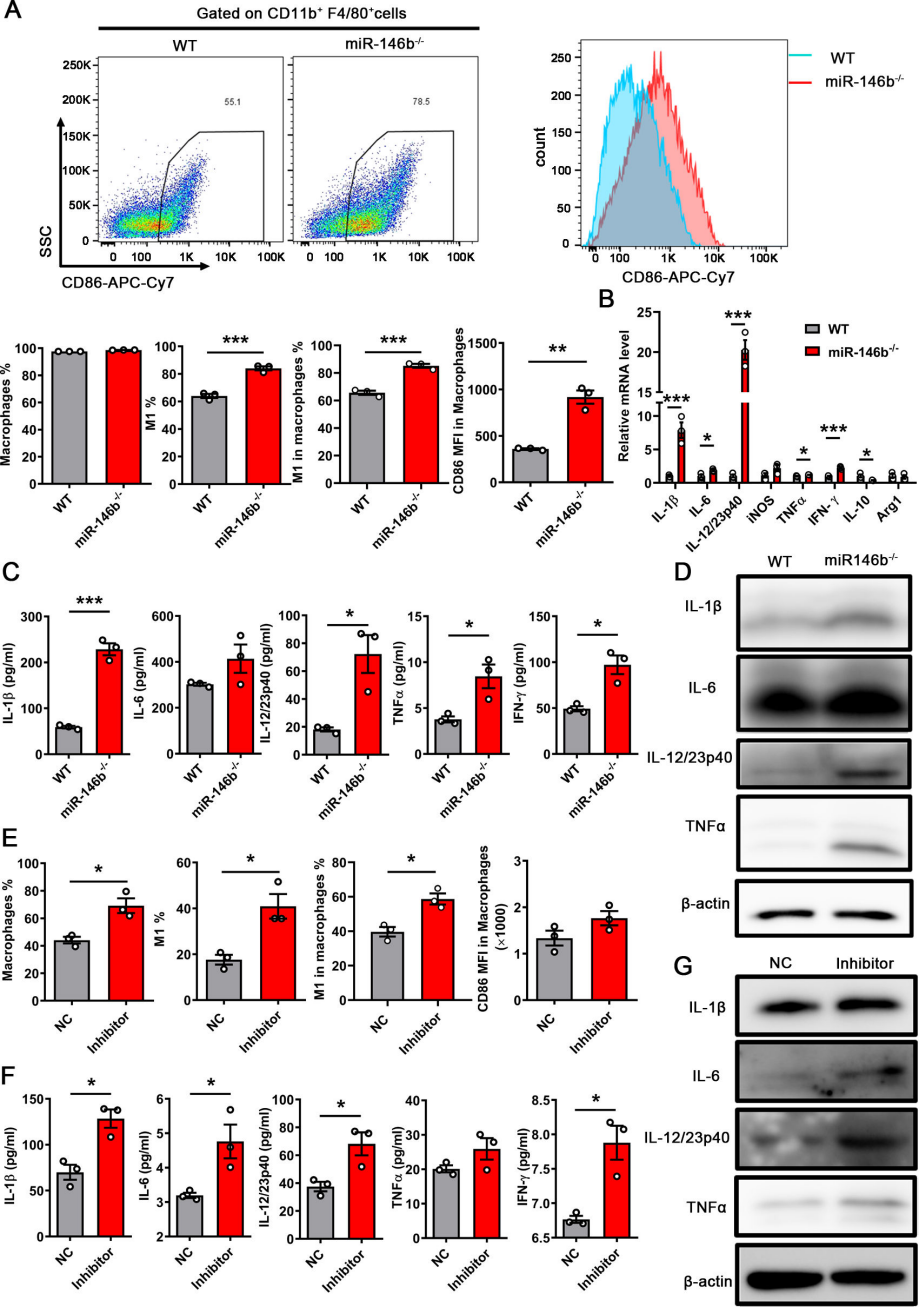
miR-146b deficiency promoted M1 macrophage differentiation. (A–D) BMDMs from WT or miR-146b^−/−^ mice were stimulated with CFT073 [multiplicity of infection (MOI) = 0.01] for 6 h. (**A**) Representative flow dot plots and MFI of CD86 in macrophages. Quantification of macrophages, M1 macrophages, M1 macrophages in macrophages, and MFI of CD86 in macrophages. (B–D) Quantitative reverse transcription PCR analysis of the mRNA levels, ELISA, and WB analysis of the protein levels of inflammatory cytokines in infected BMDMs after 6 h. (E–G) THP-1 was treated with 100 ng/mL phorbol 12-myristate 13-acetate for 48 h and then transfected with miR-146b inhibitor. After 48 h, the cells were infected with CFT073 (MOI = 0.01) for 6 h. (**E**) Quantification of macrophages, M1 macrophages, M1 macrophages in macrophages, and MFI of CD86 in macrophages of differentiated THP-1. (**F and G**) ELISA and WB analysis of the levels of inflammatory cytokines in infected THP-1 after 6 h. The data were expressed as the mean ± SEM. The *t*-test was used to compare between the groups (A–C and E–F). ^*^P < 0.05; ^**^P < 0.01; ^***^P < 0.001. All experimental data were collected in at least three independent experiments.

**FIG 5:**
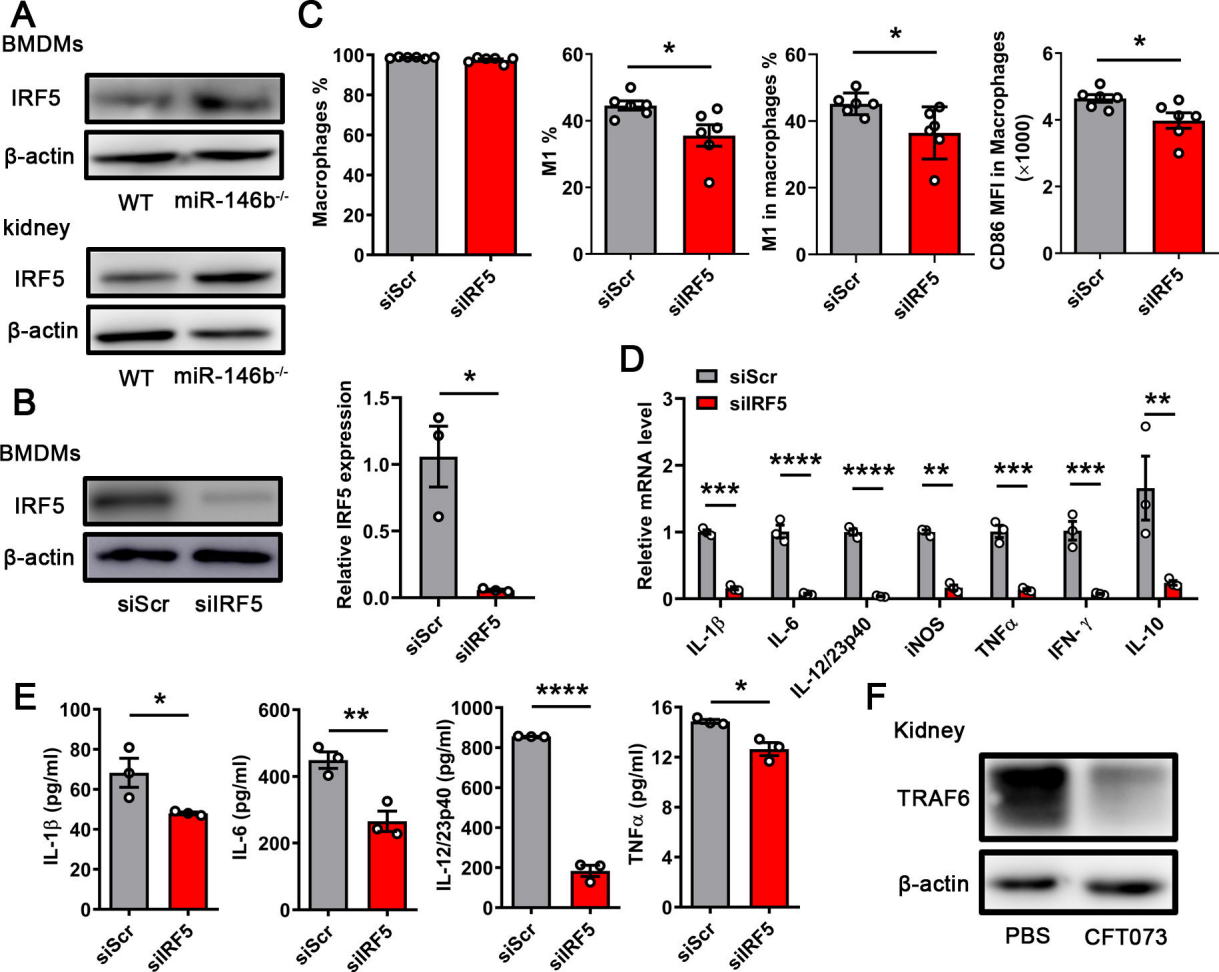
miR-146b suppressed M1 macrophages in relation to IRF5. (**A**) WB detected the expression of IRF5 in BMDMs (6 h) or the kidneys (24 h) of WT and miR-146b^−/−^ mice infected with CFT073. (**B-E**) BMDMs from WT mice transfected with siRNAs targeting IRF5 or scramble nontargeting control siRNA infected with CFT073 (multiplicity of infection = 0.01). (**B**) Detection of siRNA-induced knockdown efficiency using WB and qPCR after transfection at 48 h. (**C**) Quantification of macrophages, M1 macrophages, M1 macrophages in macrophages, and MFI of CD86 in macrophages. (**D and E**) qPCR analysis of mRNA levels and ELISA results of inflammatory cytokines in BMDMs after 6 h. (**F**) WB detected TRAF6 expression in the kidney infected with CFT073, and the control was injected with phosphate-buffered saline (PBS). The data were expressed as the mean ± SEM. The *t*-test was used to compare the groups. ^*^P < 0.05; ^**^P < 0.01; ^***^P < 0.001; ^****^P < 0.0001. All experimental data were collected in at least three independent experiments.

Our previous studies reported that IRF5 was a key transcription factor for M1 macrophage differentiation. Furthermore, IRF5 was directly targeted by miR-146b (20). Therefore, we aimed to elucidate whether miR-146b controlled the polarization of M1 macrophages through IRF5 during CFT073 infection. First, BMDMs from WT and miR-146b^−/−^ mice were stimulated with CFT073 for 6 h. The proportion of M1 macrophages was analyzed using flow cytometry, and the expression levels of some associated cytokines were examined using qPCR and ELISA. We found that BMDMs from miR-146b-deficient mice displayed significantly higher percentages of M1 macrophages than those from WT mice, and the mean fluorescence intensity (MFI) of CD86 in macrophages was also higher in miR-146b-deficient mice (Fig. 4A). This result indicated that miR-146b affected the polarization of M1 macrophages *in vitro*. However, we did not find that macrophages had enhanced differentiation into M1-type macrophages in the kidneys of infected mice after the miR-146b deletion, which might be because macrophages were mainly M1-type macrophages in the early stage of UPEC infection. Furthermore, the expression of related inflammatory factors, such as IL-1β, IL-6, IL-12, TNFα, and IFN-γ, appeared higher in BMDMs from miR-146b^−/−^ mice than from WT mice (Fig. 4B through D). When we inhibited the miR-146b expression in differentiated THP-1 cells using miR-146b inhibitors (24, 25), we found that the proportion of M1 macrophages in infected THP-1 was enhanced after inhibiting the miR-146b expression (Fig. 4E). Meanwhile, the ELISA and WB results also showed increased expression of related inflammatory factors (Fig. 4F and G). The results showed that miR-146b inhibited the differentiation of M1 macrophages during CFT073 infection. Moreover, we detected the levels of IRF5 in BMDMs and kidneys infected with CFT073 from WT and miR-146b^−/−^ mice using WB. MiR-146b deficiency increased IRF5 expression (Fig. 5A). Next, we used CFT073 to infect BMDMs in which IRF5 had been knocked down by small-interfering RNA (siRNA). We tested the transfection effect of BMDMs using WB and qPCR (Fig. 5B). The percentage and MFI of CD86 in macrophages decreased in BMDMs treated with IRF5 gene siRNA (siIRF5) (Fig. 5C). The expression of some inflammatory molecules (IL-1β, IL-6, IL-12, and TNFα) significantly decreased in the siIRF5-treated group, which differed from that in miR-146b deficiency following CFT073 infection (Fig. 5D, E, 4B and C). A previous study showed that miR-146b reduced TNF receptor-associated factor 6 (TRAF6)-dependent inflammation and improved ischemia-induced neovascularization in hypercholesterolemic conditions. We also tested TRAF6 expression in infected kidneys (24, 26). We found that TRAF6 expression in the kidney decreased after being infected by CFT073, indicating that TRAF6 might not participate in UTIs (Fig. 5F). Hence, the results suggested that miR-146b suppressed the activation of M1 macrophages by targeting IRF5 in UTIs.

### IL-10 participates in kidney injury suppression by miR-146b during UTIs

**FIG 6:**
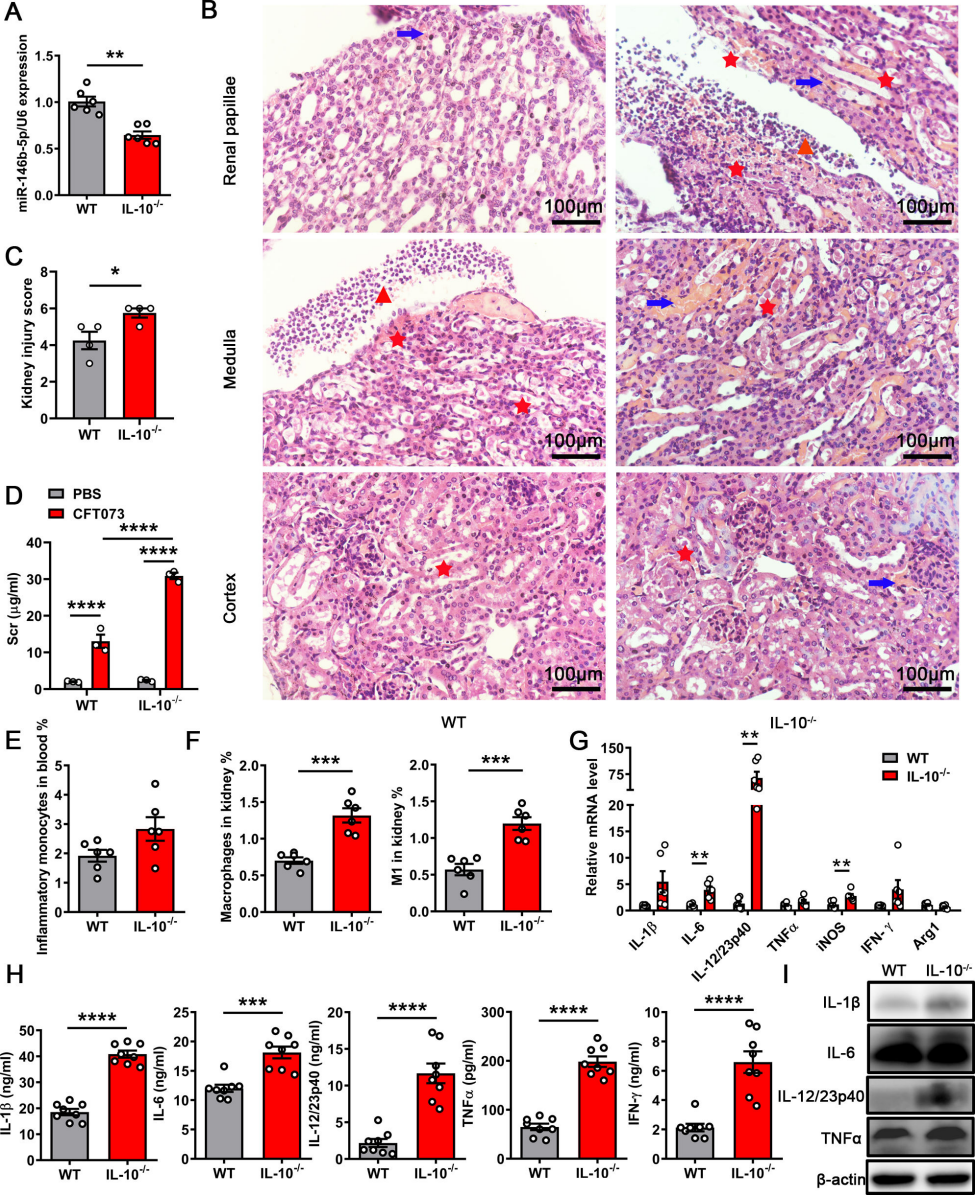
IL-10 deficiency enhances macrophage infiltration. Female WT and IL-10^−/−^ mice were inoculated intraurethrally using 1 × 10^9^ CFU of CFT073 twice at 3 h intervals. (**A**) qPCR analysis of miR-146b levels in the infected kidney after 24 h. (**B**) Representative images of H&E staining of kidney tissues after 24 h. Arrows indicate tubular casts, triangular symbols indicate inflammatory cell infiltration, and asterisks indicate renal papillae injury. Scale bar, 100 µm. (**C**) Histological scores for kidneys infected with CFT073. (**D**) Serum creatinine concentrations in WT and IL-10^−/−^ mice treated with CFT073. (**E and F**) Quantification of inflammatory monocytes in blood and macrophages in kidneys after 24 h. (**G-I**) qPCR analysis of mRNA levels, ELISA, and WB results of inflammatory cytokines in the infected kidney after 24 h. The data were expressed as the mean ± SEM. One-way analysis of variance (**D**) and *t*-test (**A, C and E-H**) were used to compare the groups. ^*^P < 0.05; ^**^P < 0.01; ^***^P < 0.001; ^****^P < 0.0001. All experimental data were collected in at least three independent experiments.

Previous studies reported that IL-10 regulated miR-146b expression (20, 25). Next, we examined whether IL-10 was involved in miR-146b-controlled kidney damage caused by CFT073. We infected WT and IL-10^−/−^ mice with CFT073 for 24 h. The qPCR analysis showed that miR-146b expression in the kidney was reduced significantly in IL-10^−/−^ mice (Fig. 6A). Pathological staining revealed that kidney injury was aggravated in IL-10-deficient mice, which were assessed using pathology scores. Tissue injury and inflammatory cell infiltration significantly increased (Fig. 6B and C; Fig. S4A). The serum creatinine and NAGL levels increased in IL-10^−/−^ mice infected with CFT073, indicating that IL-10 was related to kidney function during UTIs (Fig. 6D; Fig. S4B). Consistent with the results, the percentages of inflammatory monocytes in blood and macrophages in infected kidneys increased, as analyzed using flow cytometry (Fig. 6E and F). We also analyzed neutrophils in the blood and other immune cells in the infected kidneys, including neutrophils, NK cells, DCs, B cells, and T cells. We found that the percentages of NK cells, DCs, and T cells were significantly increased in IL-10^−/−^ mice compared with WT mice (Fig. S4C through J). The results were consistent with previous studies showing that miR-146b deficiency enhanced the activation of CD4^+^ T cells (20, 27). The mRNA expression levels of some inflammatory-associated genes (*IL-1β*, *IL-6*, *IL-12*, and *iNOS*) increased in IL-10^−/−^ mouse kidneys (Fig. 6G). The cytokine secretion of IL-1β, IL-6, IL-12, TNFα, and IFN-γ was similarly higher in the kidneys of IL-10^−/−^ mice than of WT mice (Fig. 6H). The results of WB also showed that the expression of IL-1β, IL-12, and TNFα increased in IL-10^−/−^ mice (Fig. 6I). miR-146b was regulated by IL-10 and could adjust the expression of IRF5. Hence, the expression level of IRF5 might be regulated by IL-10. However, the expression of IL-10 in BMDMs also decreased on knocking down IRF5 (Fig. 5D). This indicated other signaling pathways that regulate IL-10 expression during UTIs. These observations indicated that IL-10 induced miR-146b expression to suppress UTI-induced kidney injury.

### MiR-146b agomir improves the kidney injury caused by CFT073

**FIG 7:**
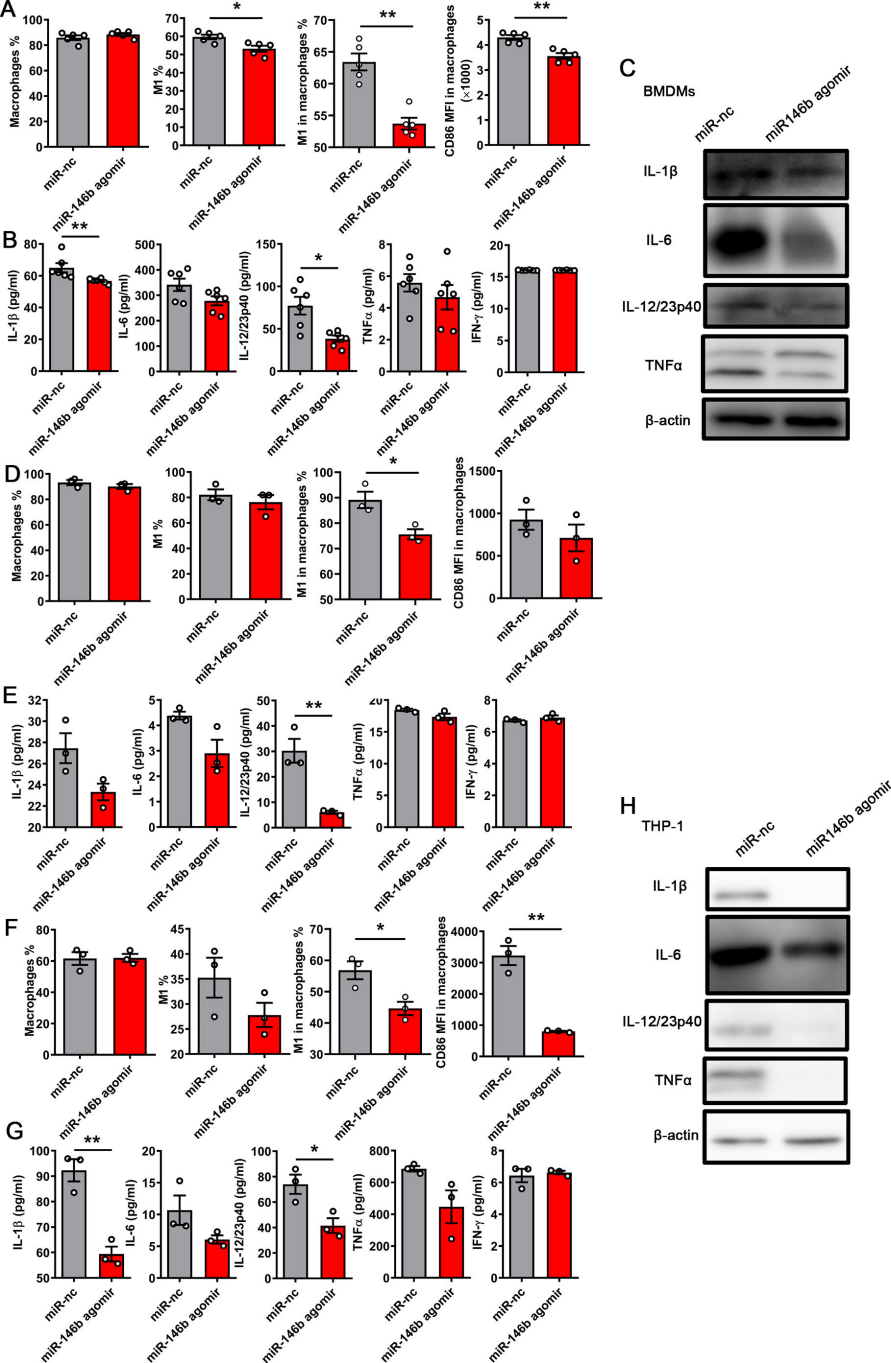
miR-146b suppressed the differentiation of M1 macrophages *in vitro*. BMDMs from C57BL/6 mice, HMDMs, and macrophages from THP-1 were transfected with miR-146b agomir (100 nM) and miR-nc (100 nM) for 48 h. Thereafter, the cells were infected with CFT073 (multiplicity of infection = 0.01) for 6 h. (**A**) Percentage of macrophages, M1 macrophages, M1 macrophages in macrophages, and MFI of CD86 macrophages in macrophages of BMDMs. (**B and C**) ELISA and WB results of inflammatory cytokines in infected BMDMs after 6 h. (**D**) Percentage of macrophages, M1 macrophages, M1 macrophages in macrophages, and MFI of CD86 macrophages in macrophages of HMDMs. (**E**) ELISA results of inflammatory cytokines in infected HMDMs after 6 h. (**F**) Percentage of macrophages, M1 macrophages, M1 macrophages in macrophages, and MFI of CD86 macrophages in macrophages of THP-1. (**G and H**) ELISA and WB results of inflammatory cytokines in infected macrophages from THP-1 after 6 h. The data were expressed as the mean ± SEM. The *t*-test was used to compare the groups. ^*^P < 0.05; ^**^P < 0.01. All experimental data were collected in at least three independent experiments.

We transfected miR-146b agomir and a scramble control into BMDMs, human monocyte-derived macrophages (HMDMs), and THP-1-derived macrophages to further investigate the function of miR-146b in regulating the orientation of M1 macrophages. After transfection for 48 h, the miR-146b expression increased, and the IRF5 expression significantly decreased in the miR-146b agomir-treated group of BMDMs (Fig. S5A and B). The cells were infected with CFT073 for another 6 h after transfection. The flow cytometry analysis showed that miR-146b agomir suppressed the differentiation of M1 macrophages in BMDMs (Fig. 7A), HMDMs (Fig. 7D), and macrophages from THP-1 (Fig. 7F). Similarly, the expression of IL-1β, IL-6, IL-12, and TNFα was reduced in BMDMs (Fig. 7B and C), HMDMs (Fig. 7E), and macrophages from THP-1 (Fig. 7G and H) treated with miR-146b agomir compared with that in the control group. Thus, the results indicated that miR-146b agomir suppressed M1 macrophage activation *in vitro* in both mice and humans.

**FIG 8:**
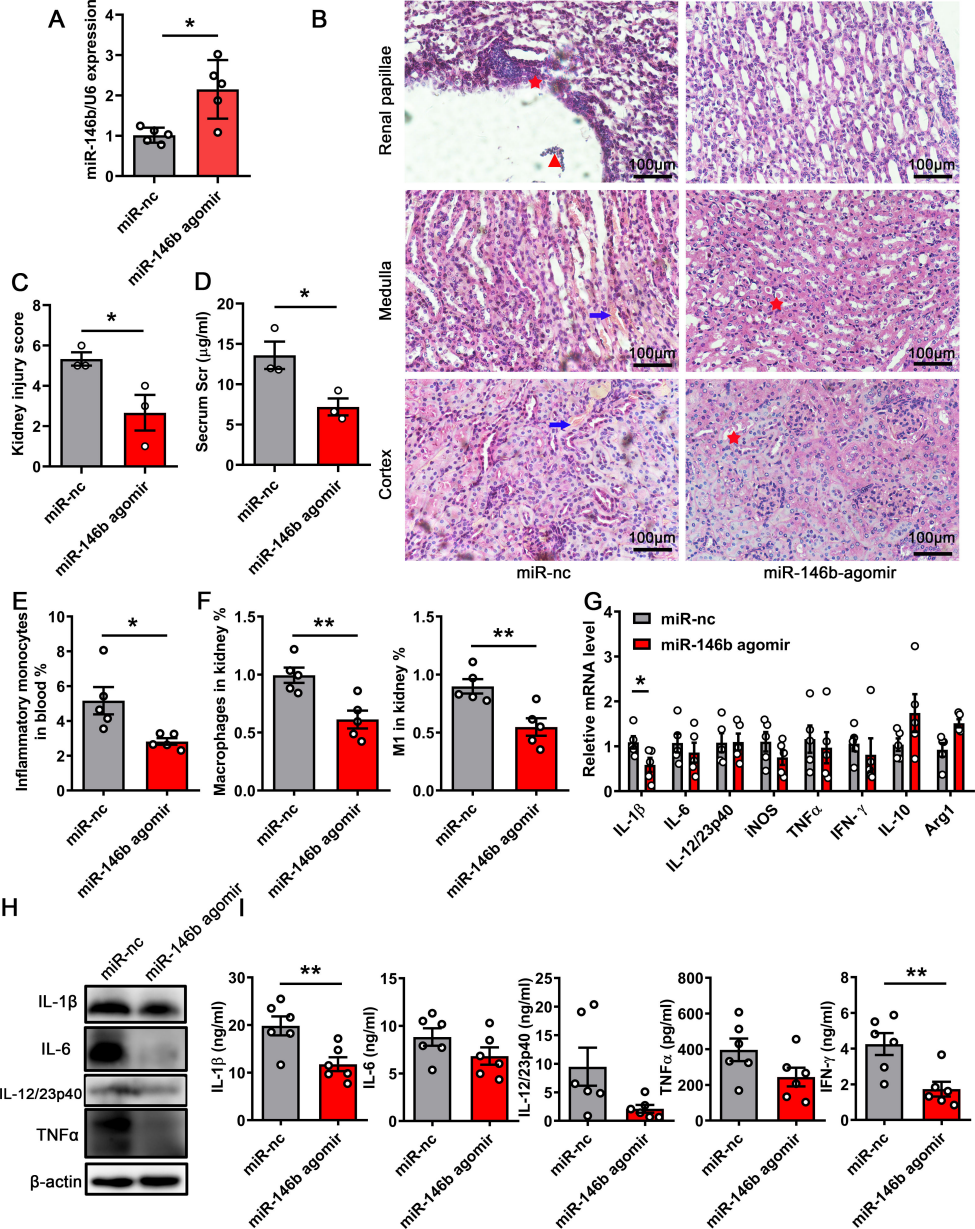
Kidney injury was ameliorated by miR-146b agomir in acute UTIs caused by CFT073. Mice were treated intravenously with miR-146b scramble or miR-146b agomir (10 nmol per mouse). The mice were then infected with CFT073 for 24 h. (**A**) Detection of miRNA transfection efficiency using qPCR in the kidney. (**B**) Representative images of H&E staining of kidney tissues after 24 h. Arrows indicate tissue hemorrhage, triangular symbols indicate inflammatory cell infiltration, and asterisks indicate renal papillae injury. Scale bar, 100 µm. (**C**) Histological scores are shown in the right panel. (**D**) Serum creatinine concentrations in mice treated with miR-146b agomir and CFT073. (**E and F**) Percentages of inflammatory monocytes in blood and macrophages in the kidneys after 24 h. (**G-I**) qPCR analysis of mRNA levels, ELISA, and WB results of inflammatory cytokines in infected kidneys after 24 h. The data were expressed as the mean ± SEM. The *t*-test was used to compare the groups. ^*^P < 0.05; ^**^P < 0.01. All experimental data were collected in at least three independent experiments.

The aforementioned results demonstrated that miR-146 played an important role in controlling the damage to infected kidneys during UTIs. Next, we evaluated the potential of miR-146 for ameliorating kidney injury in UTIs. We intravenously injected miR-146b agomir and miRNA control into mice (18, 20). Subsequently, the mice were infected with CFT073 for 24 h. The transfection effect is depicted in Fig. 8A. We found that the kidneys of miR-146b-treated mice showed less damage than those treated with the scramble control (Fig. 8B; Fig. S5C). The pathology scores were presented in Fig. 8C. The miR-146b agomir treatment also improved kidney function during UTIs (Fig. 8D; Fig. S5D). Flow cytometry analysis revealed that the percentage of M1 macrophages in the infected kidneys of mice treated with miR-146b agomir significantly decreased compared with that in control mice (Fig. 8E and F). Furthermore, we found that miR-146b agomir suppressed the expression of some inflammation-related cytokines (Fig. 8G through I). The IRF5 expression also significantly decreased in the miR-146b agomir-treated group of infected kidneys (Fig. S5E). Furthermore, we analyzed other immune cells in the kidneys, including neutrophils, NK cells, DCs, B cells, T cells, CD4^+^ T cells, and CD8^+^ T cells, using flow cytometry. The results showed that the percentages of neutrophils, DCs, and T cells were significantly reduced, but other cell types were comparable (Fig. S5F through M). Hence, the findings suggested that miR-146b could be used to treat UTIs to reduce kidney damage caused by the CFT073 infection.

## DISCUSSION

As UPEC invades a host, the immune response mobilizes to fight against the infection. However, excessive inflammatory responses cause tissue damage. Although antibiotics can effectively control the pathogenesis of UTIs, they cannot prevent kidney damage caused by excessive inflammation during UTIs (28, 29). Furthermore, long-term antibiotic treatment for recurrent infections can lead to antibiotic-resistant strains and gut microbiome disorders, posing a further threat to persistent pain in patients with UTIs (3). Thus, novel therapies to treat UTIs and prevent kidney damage caused by UTIs urgently need to be developed.

M1 macrophages have strong phagocytic abilities and can produce pro-inflammatory cytokines to strengthen the innate immune response to remove microorganisms (14, 30, 31). Our previous study found that inflammatory monocytes migrated to infected kidneys and were then polarized to M1 macrophages in the early stages of UTIs. However, excessive immune responses can lead to kidney damage during UTIs. In this study, we demonstrated that macrophage depletion alleviated kidney damage in mice infected with CFT073. The results were similar to a previous study showing that M1 macrophages were related to kidney injury caused by UTIs (15). Based on our results and a previous study, we aimed to investigate the molecular mechanisms underlying macrophage polarization to M1 macrophages and the extent of kidney damage in UTIs. Furthermore, we explored novel approaches for treating UTIs to reduce kidney damage. In this study, we found that miR-146b was clearly involved in kidney damage by regulating M1 macrophage differentiation during UTIs.

Given their unique functions, miRNAs have recently attracted much attention for treating cancer. Furthermore, miR-146b is involved in developing various cancers, including lung, liver, breast, colon, and thyroid cancers (32). MiR-146b often plays a pleiotropic role in tumors to exert tumor-promoting functions. For example, iodine can inhibit the proliferation of papillary thyroid cancer cells by downregulating miR-146b-5p in cancer cell exosomes, whereas colorectal cancer promotes white adipose tissue browning and tumor progression through exosomal miR-146b-5p in tumor cells (33, 34). Interestingly, miR-146b-5p can suppress tumor cell progression and downregulate TRAF6 by improving the chemotherapy resistance of gliomas. It also downregulates the expression of the ribonucleoprotein human antigen R to inhibit large granular lymphocytic leukemia (24, 35, 36). However, miR-146b may produce multiple effects in one type of cancer. For example, miR-146b not only promotes ovarian carcinoma cell proliferation and chemoresistance but also inhibits cell migration and invasion (32, 35). In bladder cancer, miR-146b reduces cell proliferation, migration, and invasion and induces apoptosis (37). Thus, miRNAs are often used as cancer therapeutic targets for evaluating prognosis (32, 38).

Additionally, miR-146b is involved in developing various inflammatory diseases such as hepatic schistosomiasis, psoriasis, ulcerative colitis, and myocardial infarction (18, 21, 22, 39, 40) and functions as an inhibitory factor in these diseases. Previous studies pointed out that IL-10 could promote the expression of antiinflammatory factors by inducing the expression of miR-187, miR-155, and miR-146a/b, inhibiting inflammatory responses. Also, miR-146b suppresses inflammatory responses by regulating TRAF6 and TNFα in multiple diseases. For example, miR-146b suppressed TRAF6 to inhibit the damage to the glomerular system in lupus erythematosus and rheumatoid arthritis. Furthermore, miR-146b suppressed the inflammatory response by restraining TRAF6 expression to improve neovascularization in hypercholesterolemic conditions and inhibit the growth of tumors in computed tomography-guided renal cell carcinoma. However, the incidence of hematopoietic malignancy increased considerably with miR-146b deficiency (41). In this study, the level of TRAF6 in CFT073-infected kidneys decreased, although TNFα expression increased, indicating that miR-146b regulates the inflammatory response through other signal channels. MiR-146b also targeted IRF5 expression to inhibit the activation of M1 macrophages and ameliorate colitis. Liver damage in schistosomiasis is also related to the targeted regulation of M1 macrophages by miR-146b (20–22, 26). M2 macrophages are regulated by miR-146b in fevers with thrombocytopenia syndrome, which is involved in damage repair (23). The results of this study indicated that M2 macrophages in acute UTIs were not significantly affected (data not shown). Hence, we focused on the effect of miR-146b on M1 instead of M2 macrophages. It was reported that miR-146b-5p affected kidney function in primary chronic kidney disease, which was dependent on gonadal hormone status (42).

In this study, we demonstrated that miR-146b was highly expressed in the kidneys after UTIs, and miR-146b^−/−^ mice showed aggregated kidney damage with enhanced activation of M1 macrophages. Furthermore, we found that IL-10 induced miR-146b expression, which targeted IRF5 in modulating M1 macrophage activation. Finally, the results showed that miR-146b agomir effectively reduced kidney inflammation by suppressing M1 macrophage activation after UTIs. Thus, the results suggested that miR-146b controlled renal inflammation by regulating the activation of M1 macrophages, highlighting that miR-146b might be used as a novel therapeutic target for treating UTIs to protect against renal damage caused by excessive inflammatory responses.

## MATERIALS AND METHODS

### Bacterial strain and mouse

The UPEC strain (CFT073) was kindly gifted by Professor Quan Wang from Tianjin Medical University. CFT073 was cultured at 37°C in the solid Luria–Bertani (LB) medium for 12 h. Thereafter, the selected monoclonal colonies in liquid LB were placed under static conditions for 12 h. The selected monoclonal colonies were placed in liquid LB at 37°C with shaking for 12 h to infect cells. Then, the bacteria were diluted (1:1,000) into fresh LB at 37°C until OD600 nm was between 0.6 and 0.8.

### Mouse UTI model

The WT C57BL/6 mice (WT mice) between 6 and 8 wk old were purchased from Pengyue Experimental Animal Breeding Company (Jinan, China). The IL-10-deficient (IL-10^−/−^) and miR-146b^−/−^ mice were obtained from the Nanjing University Model Animal Research Centre (Nanjing, China). All mice were bred under specific pathogen-free conditions at the animal facility of Jining Medical University. Every effort was made to minimize animal suffering and reduce the number of animals used. This study was approved by the Jining Medical University Institution Animal Care and Use Committee (No. 2021-DW-ZR-016).

The UTI mouse model was established as previously described (43). After 3 h, the anesthetized female WT mice (6‒8 wk) were inoculated twice intraurethrally with 10^9^ CFU CFT073 in 50 µL phosphate-buffered saline (PBS). The mice were euthanized 24 h post-inoculation. The blood and kidneys were removed for flow cytometry, histology, and pro-inflammatory cytokine analyses.

BMDMs were isolated from WT and miR-146b^−/−^ mice and cultured in complete Dulbecco’s modified Eagle medium (Gibco) with 10% heat-inactivated fetal calf serum (FCS) and murine granulocyte-macrophage colony-stimulating factor (10 ng/mL; Peprotech) for 6 d. PBMCs were isolated from healthy donors using Ficoll-Paque PLUS (GE Healthcare). Blood collection from healthy donors conformed to the Jining Medical University Institution Human Care and Use Committee guidelines (No. JNMC-2021-YX-004). Healthy volunteers donated their blood for this study with informed consent.

The cells were then cultured in the 1640 medium with 10% FCS and human GM-CSF (10 ng/mL; Peprotech) for 6 d. THP-1 was cultured in the 1640 medium with 10% FCS and phorbol 12-myristate 13-acetate (100 ng/mL; Sigma-Aldrich) for 48 h. Finally, we obtained BMDMs, HMDMs, and macrophages from THP-1.

BMDMs and HMDMs (5 × 10^5^/well) were seeded in 24-well plates for 12 h before UPEC treatment. Furthermore, 2 × 10^6^/well THP-1 were seeded into 6-well plates. After the corresponding treatments, the cells were treated with CFT073 [multiplicity of infection (MOI) = 0.01]. The cells and culture supernatant were analyzed using flow cytometry, quantitative reverse transcription PCR (qPCR), ELISA, and WB after 6 h.

### Flow cytometry analysis

Single-cell suspensions were generated by digestion with 1 mg/mL collagenase type IV (Gibco) and 1 mg/mL DNase I (SinoBiological) in PBS for 40 min at 37°C under mild shaking. The Fc receptors were blocked using CD16/32 (14-0161-82; BioLegend) for 10 min. Single-cell suspensions were then incubated with the following antibodies purchased from BioLegend: FITC antimouse CD11b (101206), PE antimouse F4/80 (123110), APC-Cy7 antimouse CD86 (105030), APC antimouse CD206 (141708), PE antimouse Ly6G (127608), APC antimouse Ly6C (128016), PE antimouse NK1.1 (108708), FITC antimouse B220 (103206), PE antimouse CD3 (100206), FITC antimouse CD4 (100406), APC antimouse CD8 (100712), and BV421 antimouse CD45 (103134). HMDMs were stained with BV421 antihuman CD68 (333828), FITC antihuman CD14 (982502), APC antihuman CD86 (374208), and PE antihuman CD206 (321106). The cells were analyzed using the FACSCanto Flow Cytometer (BD Biosciences).

### H&E and immumohistochemical staining

The kidneys were fixed with 4% paraformaldehyde for at least 24 h. Thereafter, the fixed tissue was embedded in paraffin and cut into 5 µm sections. The slides were stained with H&E. Renal NAGL expression was demonstrated by immunohistochemistry staining following the manufacturer’s protocol. Rabbit antimouse monoclonal antibody against NAGL (1:2,000; abcam) was used. The results of H&E staining were assessed using a 6-point scale, in which 0, 1, 2, and 3 indicated normal, mild, moderate, and severe histological lesions (pathological damage was mainly located within the medulla and the cortical-medullar junction); meanwhile, 4, 5, and 6 indicated mild, moderate, and severe histological lesions (pathological damage was mainly located in more parts of the kidney) (44–46).

### RNA extraction and qPCR

Mouse BMDMs and HMDMs were infected with CFT073 (MOI = 0.01) for 6 h. Meanwhile, the mice were infected with CFT073 for 24 h, and kidneys were removed for qPCR. RNA was extracted using RNAiso Plus (TaKaRa). Complementary DNAs were obtained using a reverse transcription kit (Vazyme Biotech). qPCR was performed using a SYBR Green PCR Master Mix (Vazyme Biotech) using a LightCycler480 (Roche). GAPDH was used as the endogenous control and quantified using the comparative critical threshold cycle 2^−∆∆Ct^ method. U6 was used as an endogenous control for miRNA. The primers used in the study are listed in Table 1.

**TABLE 1:** All primers and IRF5 siRNA sequences used in this study

Primer	Sequence (5′–3′)	Description
mIL-1β-FP	GAAATGCCACCTTTTGACAGTG	For mouse IL-1β qPCR
mIL-1β-RP	TGGATGCTCTCATCAGGACAG	For mouse IL-1β qPCR
mIL-6-FP	TAGTCCTTCCTACCCCAATTTCC	For mouse IL-6 qPCR
mIL-6-RP	TTGGTCCTTAGCCACTCCTTC	For mouse IL-6 qPCR
mIL-12-FP	AGACATGGAGTCATAGGCTCTG	For mouse IL-12 qPCR
mIL-12-RP	CCATTTTCCTTCTTGTGGAGCA	For mouse IL-12 qPCR
mTNFα-FP	GCCACCACGCTCTTCTGTCT	For mouse TNFα qPCR
mTNFα-RP	GGTCTGGGCCATAGAACTGATG	For mouse TNFα qPCR
miNOS-FP	GTTCTCAGCCCAACAATACAAGA	For mouse iNOS qPCR
miNOS-RP	GTGGACGGGTCGATGTCAC	For mouse iNOS qPCR
mIFNγ-FP	ATGAACGCTACACACTGCATC	For mouse IFN-γ qPCR
mIFNγ-RP	CCATCCTTTTGCCAGTTCCTC	For mouse IFN-γ qPCR
mIL-10-FP	GCCAGAGCCACATGCTCCTA	For mouse IL-10 qPCR
mIL-10-RP	GATAAGGCTTGGCAACCCAAGTAA	For mouse IL-10 qPCR
mArg1-FP	TGTCCCTAATGACAGCTCCTT	For mouse Arg1 qPCR
mArg1-RP	GCATCCACCCAAATGACACAT	For mouse Arg1 qPCR
mGAPDH-FP	CTAGAGAGCTGACAGTGGGTAT	For mouse GAPDH qPCR
mGAPDH-RP	AGACGACCAATGCGTCCAAA	For mouse GAPDH qPCR
mIRF5-FP	GGTCAACGGGGAAAAGAAACT	For mouse IRF5 qPCR
mIRF5-RP	CATCCACCCCTTCAGTGTACT	For mouse IRF5 qPCR
hIRF5-FP	GGGCTTCAATGGGTCAACG	For human IRF5 qPCR
hIRF5-RP	GCCTTCGGTGTATTTCCCTG	For human IRF5 qPCR
hGAPDH-FP	GGAGCGAGATCCCTCCAAAAT	For human GAPDH qPCR
hGAPDH-RP	GGCTGTTGTCATACTTCTCATGG	For human GAPDH qPCR
m-miR-146b-5p	ACACTCCAGCTGGGTGAGAACTGAATTCCA	For miR-146b-5p qPCR
m-miR-146b-3p	GCCCTAGGGACTCAGTTCTGGT	For miR-146b-3p qPCR
m-miR-146a-5p	TGAGAACTGAATTCCATGGGTT	For miR-146a-5p qPCR
m-miR-146a-3p	CCTCTGAAATTCAGTTCTTCAG	For miR-146a-3p qPCR
U6-FP	CTCGCTTCGGCAGCACA	For U6 qPCR
U6-RP	AACGCTTCACGAATTTGCGT	For U6 qPCR
IRF5-Mus-1524 sense	GCAGUUUAAAGAGCUUCAUTT	
IRF5-Mus-1524 antisense	AUGAAGCUCUUUAAACUGCTT	
Negative control sense	UUCUCCGAACGUGUCACGUTT	
Negative control antisense	ACGUGACACGUUCGGAGAATT	

### Clod liposome treatment

Before 24 h of infection, 200 µL of control liposomes or Clod liposomes (FormuMax) was administered intravenously to eliminate macrophages (14). Next, the mice were used to generate the UTI model.

### RNA interference

Scrambled siRNA and mouse siIRF5 were synthesized by GenePharma (Shanghai, China). The siRNA was transfected into BMDMs using Lipofectamine 2000 (Invitrogen). The cells were collected 48 h post-transfection and infected with CFT073. The siRNA sequences are listed in Table 1.

### Enzyme-linked immunosorbent assay

The inflammatory cytokines (IL-1β/IL-6/IL-12p40/IFN-γ/TNFα) in the supernatants from infected BMDMs, HMDMs, and macrophages from THP-1 and homogenized kidneys (one whole kidney was homogenized and lysed in 1 mL of lysate) were measured using mouse ELISA kits (BioLegend) and human ELISA kits (Neobioscience).

### WB analysis

Whole-cell lysates were obtained using RIPA lysis buffer containing complete protease inhibitors (Beyotime Biotechnology). The protein concentration was assayed using a BCA Protein Assay Kit (Beyotime Biotechnology). The antibody against mouse IRF5 (1:1,000; CST), antibody against β-actin (1:1,000; Beyotime Biotechnology), antibody against IL-1β (1:1,000; CST), antibody against IL-6 (1:1,000; Santa Cruz), antibody against IL-12 (1:1,000; Santa Cruz), antibody against TNFα (1:1,000; CST), antibody against mouse TRAF6 (1:1,000; Abcam), horseradish peroxidase (HRP)-conjugated antirabbit immunoglobulin G (IgG) (1:5,000; Beyotime Biotechnology), and HRP-conjugated antimouse IgG (1:5,000; Beyotime Biotechnology) were used to detect the expression of IRF5, IL-1β, IL-6, IL-12, TNFα, TRAF6, and β-actin. The signals were detected using the Immobilon Western Chemiluminescent HRP Substrate (Wanleibio) and analyzed using the GE Amersham Imager 600 machine.

### MiR-146b agomir and inhibitor transfection *in vitro* or *in vivo*

BMDMs, HMDMs, and macrophages from THP-1 were seeded in 24-well plates at a density of 5 × 10^5^ cells/well. The cells were treated with a miR-146b-5p agomir, a miR-146b-5p inhibitor, and a negative control (100 nM) (RiboBio) using Lipofectamine 2000 reagent (Invitrogen) for 48 h following the manufacturer’s protocols. THP-1 was transfected using the Lipofectamine RNAiMAX reagent (Invitrogen). MiR-146b-5p agomir and a negative control (10 nmol per mouse) were transfected into C57BL/6 mice (18, 20). The mice were treated with CFT073 for UTIs.

### Statistical analysis

All data were analyzed using SPSS Statistics (SPSS Software Inc., IL, USA) and expressed as mean ± SEM. The *t*-test was used for the two groups, and one-way analysis of variance was used to analyze the four groups. A *P* value <0.05 indicated a statistically significant difference (*P <  0.05; **P < 0.01; ***P < 0.001;****P < 0.0001). All experimental data were collected in at least three independent experiments.

## Data Availability

All original data are available from the corresponding authors upon reasonable request.

## SUPPLEMENTAL MATERIAL

Supplemental figures mbio.02094-23-s0001.pdf Figures S1 to S5.

ASM does not own the copyrights to Supplemental Material that may be linked to, or accessed through, an article. The authors have granted ASM a non-exclusive, world-wide license to publish the Supplemental Material files. Please contact the corresponding author directly for reuse.
