# Supplementary material for: MicroRNA-146b protects kidney injury during urinary tract infections by modulating macrophage polarization
Source: mBio. 2023 Nov 1;14(6):e02094-23. doi: 10.1128/mbio.02094-23 (PMC10870822; doi:10.1128/mbio.02094-23)
Supplement: Supplemental figures — Figures S1 to S5. [file mbio.02094-23-s0001.pdf]

# Suppmental materials

**MicroRNA-146b Protects Kidney Injury during Urinary Tract Infections by Modulating Macrophage Polarization**

Changying Wang[a, b], Hongyan Cheng [a, c], Fenglian Yan [a, b], Hui Zhang [a, b], Junfeng Zhang [a, b], Chunxia Li [a, b], Mingsheng Zhao [a, b], Dongmei Shi [d], Huabao Xiong [a, b]

[a] Institute of Immunology and Molecular Medicine, Jining Medical University, Jining, China

[b] Jining Key Laboratory of Immunology, Jining Medical University, Jining, China

[c] Cheeloo College of Medicine, Shandong University, Jinan, China

[d] Department of Dermatology and Laboratory of Medical Mycology, Jining No. 1 People's Hospital, Shandong Province, Jining, China.

**Correspondence** Huabao Xiong, Jining Medical University, No. 133 Hehua Road, Jining 272067, Shandong, China.

**Email**: xionghbl@yahoo.com

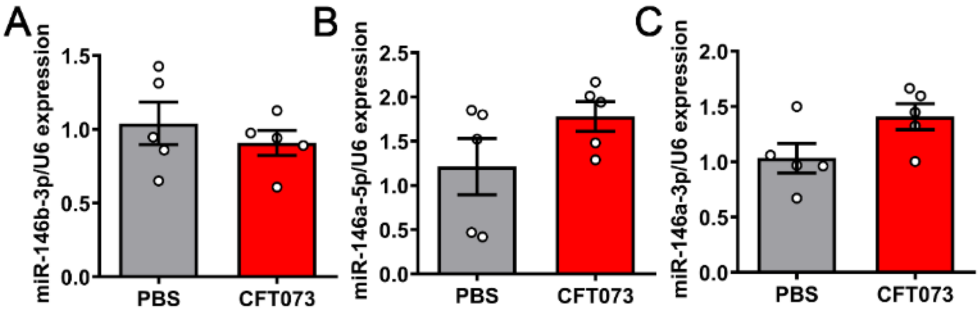


**Figure S1.** MiR-146a was least related to UPEC and induced acute UTIs. Female C57BL/6 mice were inoculated intraurethrally using $1\times10^9$ CFU of CFT073 twice at 3-h intervals. (A–C) qPCR analysis of mRNA levels for miR-146b-3p, miR-146a-3p, and miR-146a-5p in infected kidney. The data was expressed as mean ± SEM. The *t*-test was used to compare between the groups. All experimental data were performed in at

least three independent experiments.

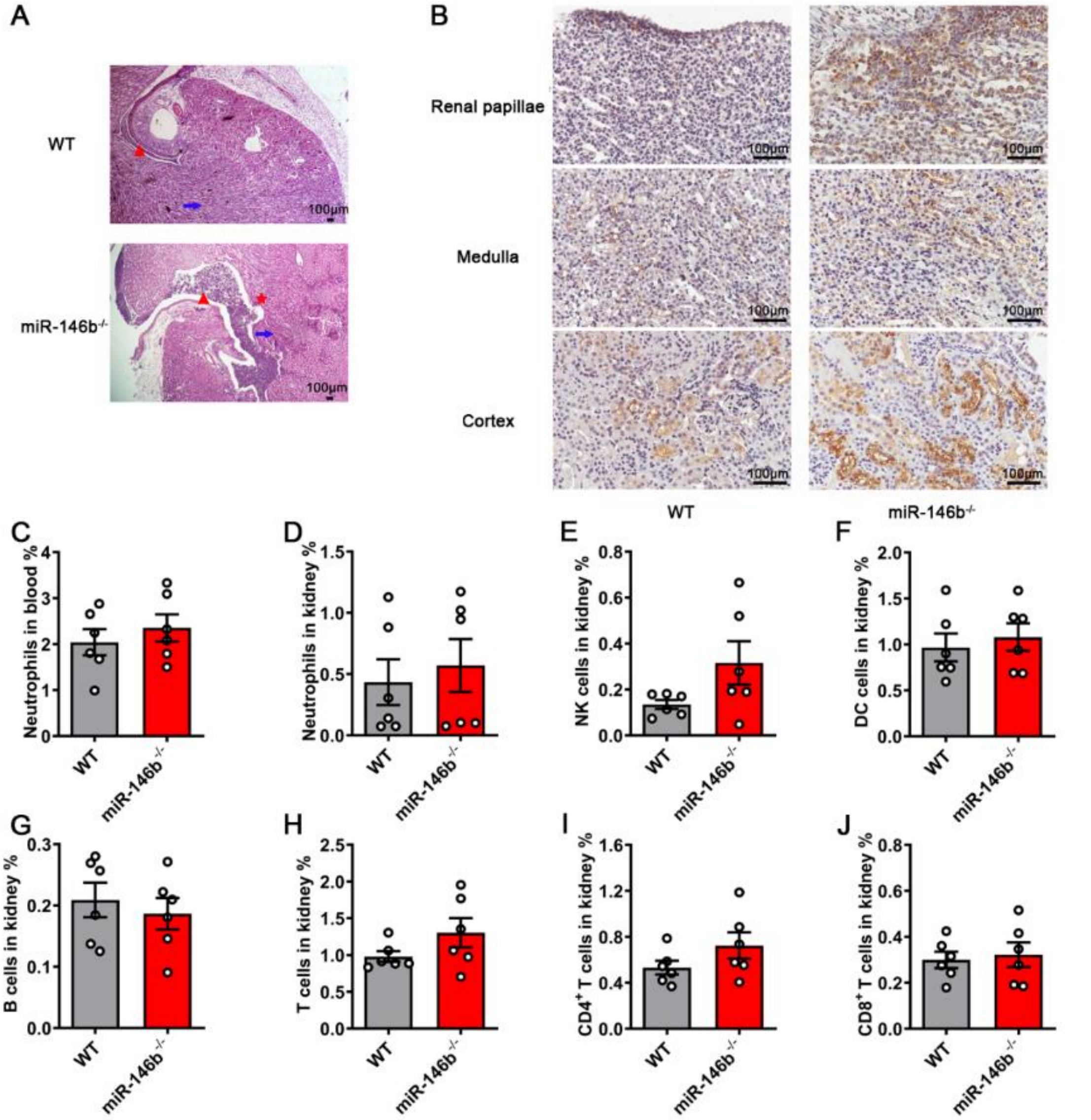


**Figure S2.** Kidney injury and various immune cells in the kidney of female WT and miR-146b$^{-/-}$ mice infected with CFT073. Female WT and miR-146b$^{-/-}$ mice were inoculated intraurethrally using $1\times10^9$ CFU of CFT073 twice at 3-h intervals. (A) Representative images of H&E staining of kidney tissues after 24 h. Arrows indicate serious hemorrhage, triangular symbols indicate inflammatory cell infiltration, and asterisks indicate renal papillae injury. Scale bar, 100 μm. (B) NAGL immunohistochemical analysis of kidney sections from WT and miR-146b$^{-/-}$ mice. Scale bars, 100 μm. (C–J) Quantification of neutrophils in blood and neutrophils, NK cells, DCs, B cells, T cells, CD4$^+$ T cells, and CD8$^+$ T cells in the kidney after 24 h. The

data was expressed as mean ± SEM. The *t*-test was used to compare between the groups. All experimental data were performed in at least three independent experiments.

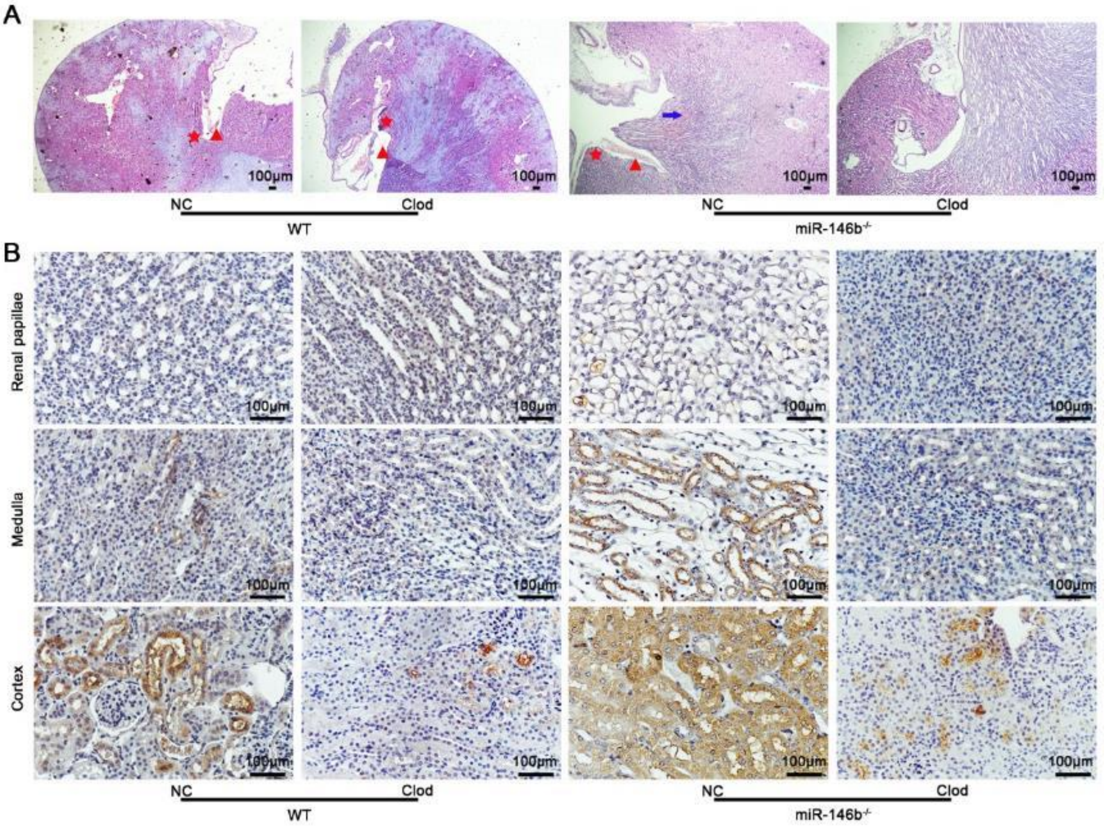


**Figure S3.** Expression of NAGL in WT and miR-146b$^{-/-}$ mice after treatment with CFT073 and Clod liposome. Female WT and IL-10$^{-/-}$ mice were treated with Clod liposome and inoculated intraurethrally using $1\times10^9$ CFU of CFT073 twice at 3-h intervals. (A) Representative images of H&E staining of kidney tissues. Arrows indicate serious hemorrhage, triangular symbols indicate inflammatory cell infiltration, and asterisks indicate renal papillae injury, protein casts. Scale bar, 100 μm. (B) NAGL immunohistochemical analysis of kidney sections from mice treated with CFT073 and Clod liposome. Scale bars, 100 μm.

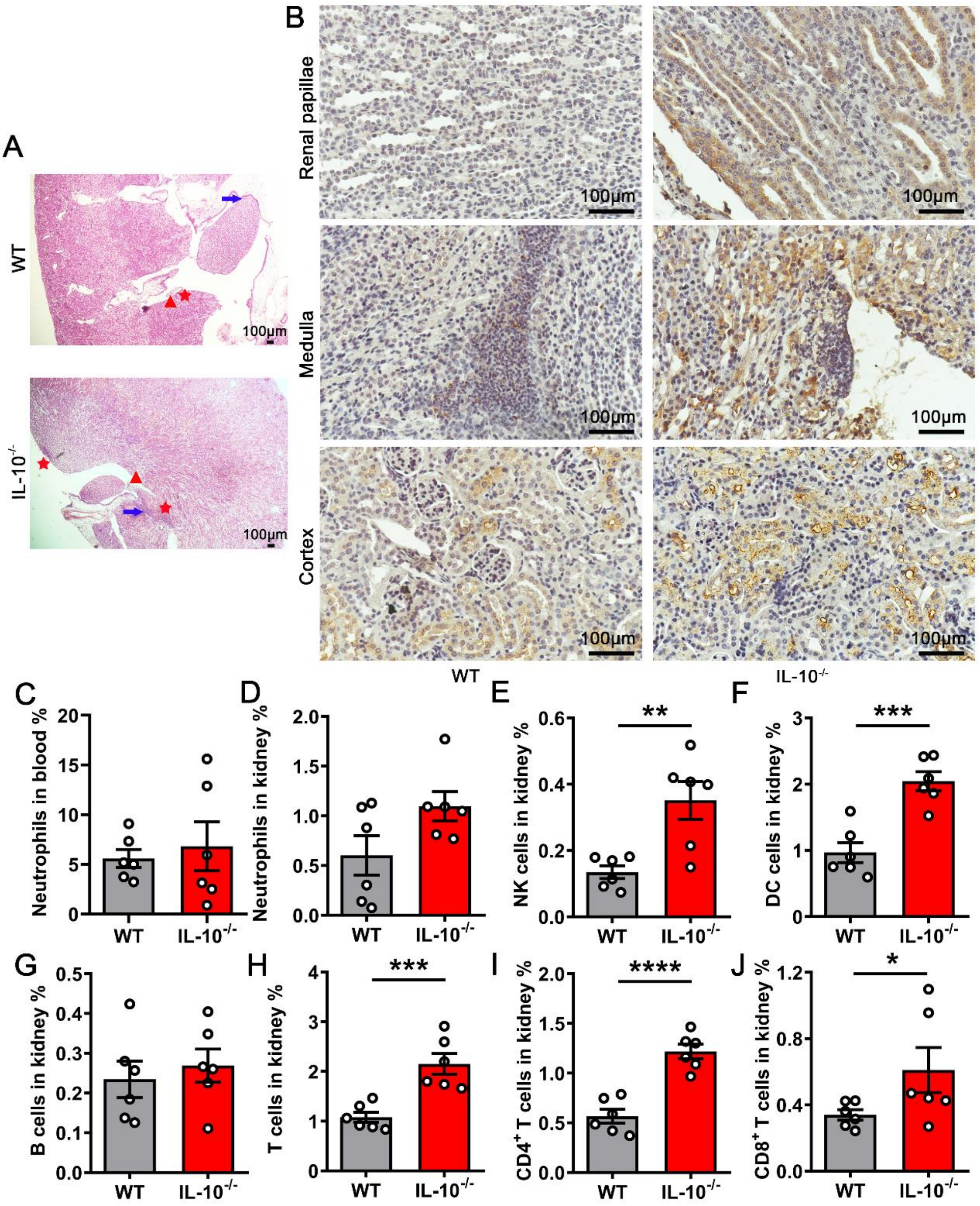


**Figure S4.** Changes of renal function and various immune cells in the kidneys of female WT and IL-10$^{-/-}$ mice infected with CFT073. Female WT and IL-10$^{-/-}$ mice were inoculated intraurethrally using $1\times10^9$ CFU of CFT073 twice at 3-h intervals. (A) Representative images of H&E staining of kidney tissues after 24 h. Arrows indicate tubular casts, triangular symbols indicate inflammatory cell infiltration, and asterisks indicate renal papillae injury. Scale bar, 100 μm. (B) NAGL immunohistochemical analysis of kidney sections from WT and IL-10$^{-/-}$ mice. Scale bars, 100 μm. (C–J) Quantification of neutrophils in blood and neutrophils, NK cells, DC cells, B cells, T

cells, CD4$^+$ T cells, and CD8$^+$ T cells in the kidney after 24 h. The data was expressed as mean ± SEM. The *t*-test was used to compare between the groups. $^*P < 0.05$; $^{**}P < 0.01$. All experimental data were performed in at least three independent experiments.

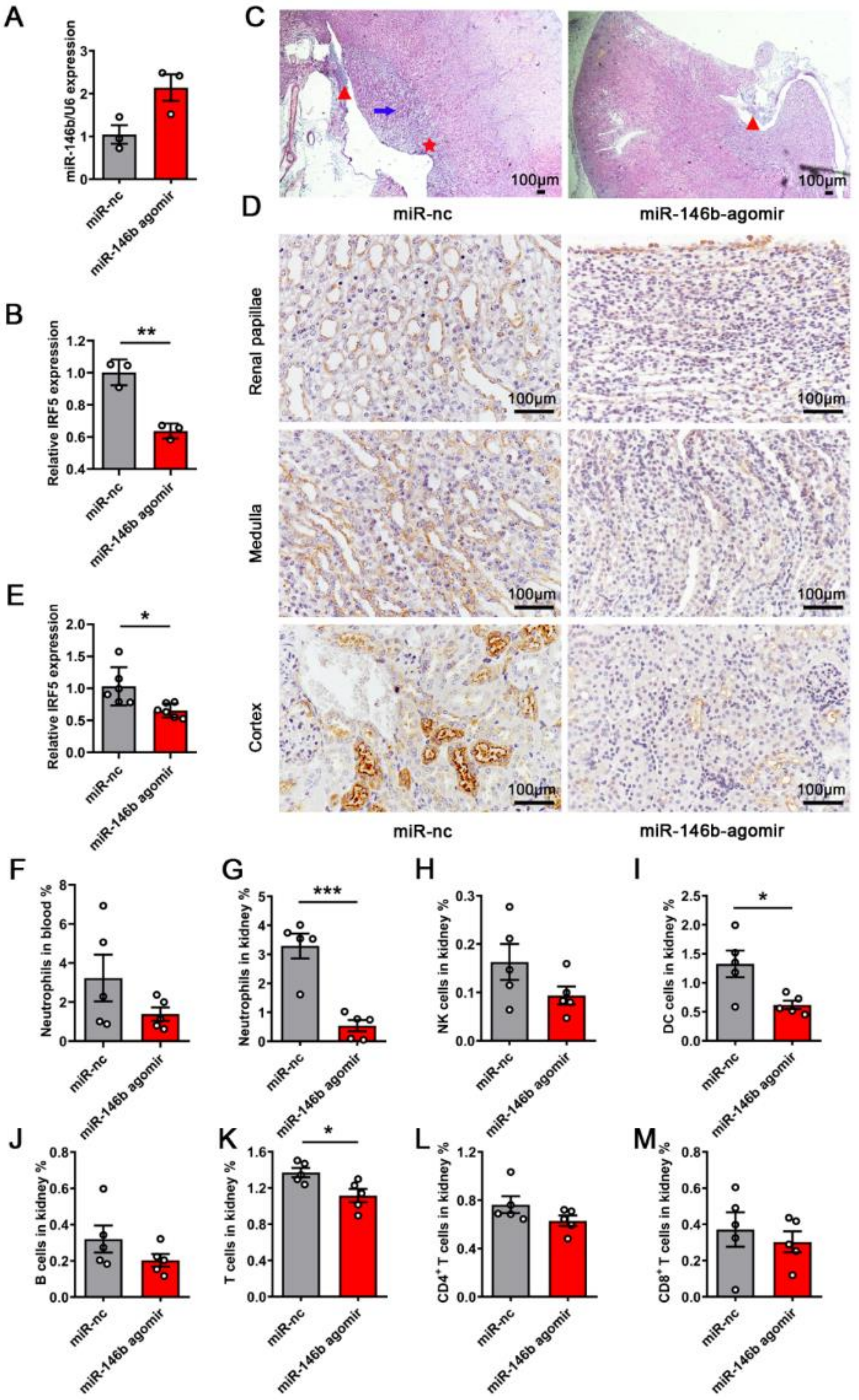


**Figure S5.** Changes in renal function and various immune cells in mice kidneys treated with miR-146b agomir and infected with CFT073. (A and B) qPCR analysis of miRNA

transfection efficiency and the expression of miR-146b and IRF5 in the BMDMs. (C-L) Mice were treated intravenously with miR-146b scramble or miR-146b agomir (10 nmol per mouse), and then they were infected with CFT073 for 24 h. (C) Representative images of H&E staining of kidney tissues after 24 h. Arrows indicate tissue hemorrhage, triangular symbols indicate inflammatory cell infiltration, and asterisks indicate renal papillae injury. Scale bar, 100 μm. (D) NAGL immunohistochemical analysis of kidney sections from miR-146b agomir-treated mice. Scale bars, 100 μm. (E) qPCR analysis of IRF5 expression level in kidney. (F–M) Quantification of neutrophils in blood and neutrophils, NK cells, DCs, B cells, T cells, $CD4^+$ T cells, and $CD8^+$ T cells in the kidney after 24 h. The data was expressed as mean ± SEM. The *t*-test was used to compare between the groups. All experimental data were performed in at least three independent experiments.
